# Radiation loss of planar surface plasmon polaritons transmission lines at microwave frequencies

**DOI:** 10.1038/s41598-017-06454-y

**Published:** 2017-07-21

**Authors:** Zhixia Xu, Shunli Li, Xiaoxing Yin, Hongxin Zhao, Leilei Liu

**Affiliations:** 1 State Key Laboratory of Millimeter Waves, Southeast University, Nanjing, 210096 China; 2 Synergetic Innovation Center of Wireless Communication Technology, Southeast University, Nanjing, 210096 China; 3 School of Electronic Science and Engineering, Nanjing University of Posts and Telecommunications, Nanjing, 210003 China

## Abstract

Radiation loss of a typical spoof surface plasmon polaritons (SSPPs) transmission line (TL) is investigated in this paper. A 325 mm-long SSPPs TL is designed and fabricated. Simulated results show that radiation loss contributes more to transmission loss than dielectric loss and conductor loss from 2 GHz to 10 GHz. Radiation loss of the SSPPs TL could be divided into two parts, one is caused by the input mode converter, and the other is caused by the corrugated metallic strip. This paper explains mechanisms of radiation loss from different parts, designs a loaded SSPPs TL with a series of resistors to absorb electromagnetic energy on corrugated metallic strip, and then discriminates radiation loss from the input mode converter, proposes the concept of average radiation length (ARL) to evaluate radiation loss from SSPPs of finite length, and concludes that radiation loss is mainly caused by corrugated structure of finite length at low frequency band and by the input mode converter at high frequency band. To suppress radiation loss, a mixed slow wave TL based on the combination of coplanar waveguides (CPWs) and SSPPs is presented. The designed structure, sample fabrication and experimental verification are discussed.

## Introduction

Surface plasmon polaritons (SPPs) usually exist in the optical frequency region^1^ but periodic structures can help mimic SPPs at lower frequencies^2^. Using periodic structures, many transmission lines (TLs) are designed to support the propagation of spoof SPPs (SSPPs) in the microwave and THz ranges^3–11^. Meanwhile, based on SPP TLs, many researches on circuit components have been conducted^12–17^. These SPP TLs reduce electromagnetic (EM) field distribution in substrates and thus suppress dielectric loss effectively. Focusing on this phenomenon, Zhang, H. C. *et al*.^10^ investigated the low-loss characteristic of SPP TLs without taking radiation loss into considerations. Kianinejad, A. *et al*. thought that radiation loss from corrugated metallic strip of finite length was negligible and attributed radiation loss to mode converters^18^. Previous studies claimed that radiation from SPP TLs could be ignored at microwave frequencies because SPPs are bounded tightly around plasmonic metamaterials^19–21^. Nevertheless, it is uncertain whether all the energy is bounded perfectly. Therefore, investigating radiation from SPP TLs seems necessary.

In this paper, a typical SPP TL is manufactured and its radiation is investigated by simulations and experiments. An obvious radiation phenomenon from the SPP TL is found. Both simulated results and measured results indicate that radiation is the main source of loss at microwave frequencies in this case. More than 25% of total energy input the SPP TL is radiated out at the full frequency band, and over 30% of input energy is radiated out at low frequency band. Compared to dielectric loss and conductor loss, radiation loss is the dominant loss at this frequency band.

To explain the mechanism of radiation from SPP TLs, this paper analyzes the radiation from the input mode converter and that from corrugated metallic strip of finite length separately, by regarding the SPP TL as a kind of surface wave antenna^22^. The field distribution indicates that radiation from the input mode converter resembles a pair of planar horn antennas^23^ with difference beam. This paper designs a loaded SSPPs TL with a series of resistors with gradual value to calculate radiation loss from the input mode converter. By observing current distribution on an SPP TL, we deduce that surface currents towards the direction of propagation are the main source of radiation. Single corrugated metallic strip of finite length could be regarded as a long wire antenna. A radiation model is proposed to validate our explanation, predicting numbers of radiation lobes well. Furthermore, based on the effective radiation section (ERS) theory^24, 25^, the concept of average radiation length (ARL) is proposed to estimate the relationship between radiation loss and frequency because ARL is proportional to radiation loss. It is found that at low frequency band, the corrugated metallic strip of finite length is the key factor of radiation loss; at high frequency band, the input mode converter is the main source of radiation loss.

To suppress radiation from planar SPP TLs at microwave frequencies, we designed, fabricated and measured a mixed slow-wave TL. The simulated and measured S-parameters are presented. Radiation loss of the mixed TL is much less than that of the traditional SPP TL. Hopefully, the structure could find applications in low-loss and slow-wave TL designs.

## Results

### Total radiation loss

**Figure 1:**
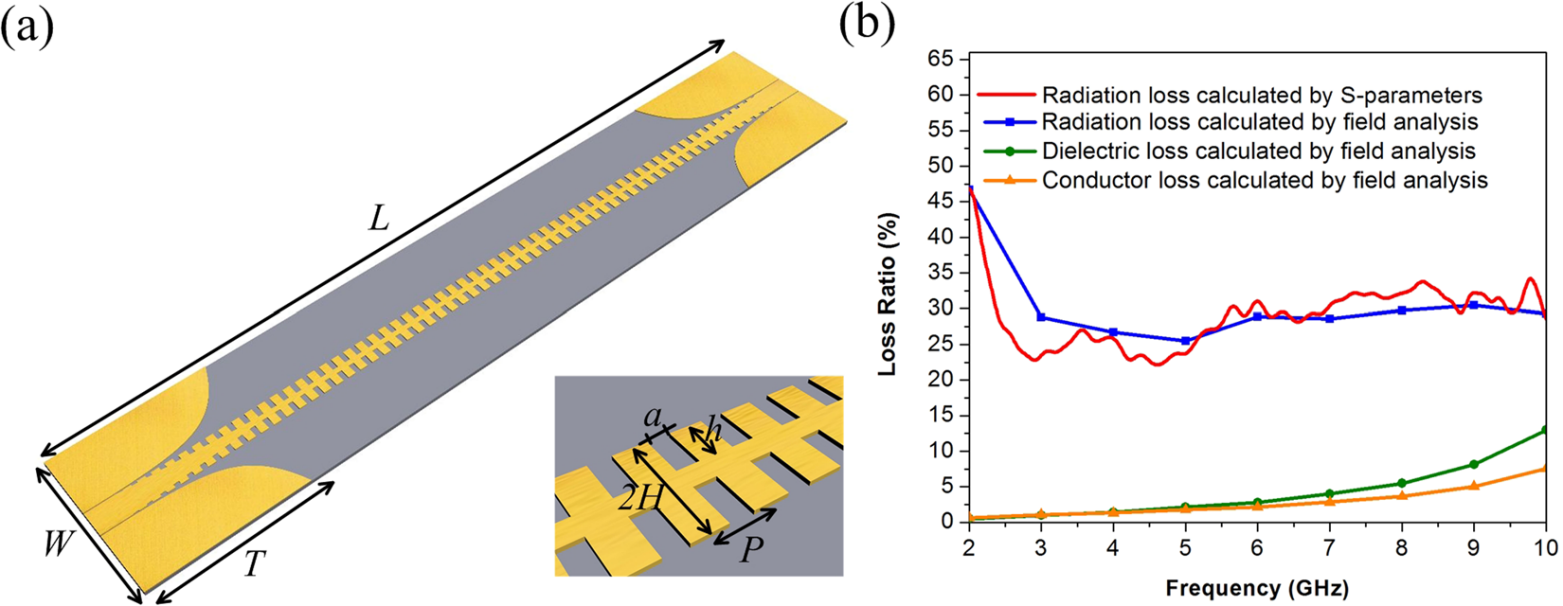
(**a**) The configuration of the SPP TL under study in which *L* = 325 mm, *W* = 60.8 mm, *T* = 60 mm, thickness of the F4B substrate is 0.5 mm and thickness of the copper conductor is 0.035 mm. The geometry of the unit cell in which *P* = 5 mm, *a* = 2 mm, *h* = 4 mm, *H* = 5 mm. (**b**) Simulated energy losses from different parts.

To investigate radiation loss, a typical SPP TL^8^ is designed in this paper. Figure 1(a) is the TL under study and the inset picture shows the unit structure of the SPP TL. The configuration and dimensions are presented in the caption. The single layer structure is 0.035 mm thick copper printed on one side of a 0.5 mm thick F4B substrate whose loss tangent is 0.003. Three kinds of energy loss, including conductor loss, dielectric loss and radiation loss, can be calculated separately by CST Microwave Studio, as shown in Fig. 1(b).

In this section, two different methods are used to estimate radiation loss. In the first method, radiation loss ratio *R*_*RL*_ is calculated by using Eq. (1) based on S-parameters in which the conductor loss ratio *R*_*CL*_ and the dielectric loss ratio *R*_*DL*_ are incorporated into the calculation.

1 $${R}_{RL}=1-{|{S}_{11}|}^{2}-{|{S}_{21}|}^{2}-{R}_{CL}-{R}_{DL}$$

In the second method, radiation loss is calculated by far-field analysis and the conductor and dielectric losses are also taken into account. The results calculated from the two methods are shown in Fig. 1(b), and it is evident that radiation loss is comparable to both dielectric loss and conductor loss, although dielectric loss and conductor loss become relatively larger when frequency increases. The difference between results calculated from S-parameters and that calculated from far field analysis is due to the existence of higher order modes which is relevant to the port size setting in simulation models.

It is difficult to discriminate between radiation from the input mode converter and that from the corrugated metallic strip by both simulations and measurements because the SPPs TL, as a whole structure, is consisted of converters and corrugated metallic strip. When the SPP TL is regarded as a surface wave antenna, the total radiation loss *R*_*RL*_ is consisted of *R*_*F*_ the radiation loss from the input mode converter, and *R*_*T*_ the radiation loss from corrugated metallic strip of finite length^22^.

2 $${R}_{RL}={R}_{F}+{R}_{T}$$

### Radiation loss from the input mode converter

**Figure 2:**
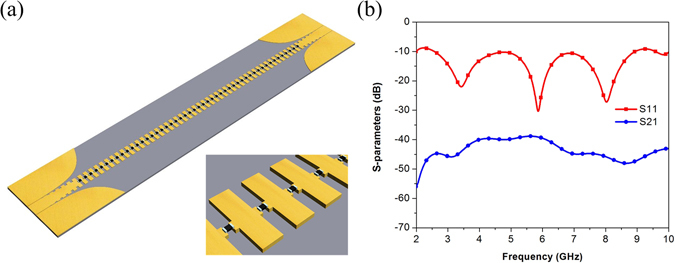
(**a**) The configuration of loaded TL with resistors. (**b**) The simulated S-parameters of loaded TL with resistors.

The input mode converter transforms the EM field from the mode of coplanar waveguides (CPW) into the mode of the SSPPs, and it can be regarded as a pair of CPW flaring horn antennas^23^ split by the middle corrugated strip exhibiting a difference beam radiation characteristic. In this paper, a loaded SPP TL with resistors of gradual value from 10 ohms to 100 ohms is designed to estimate radiation loss brought by the input mode converter. The configuration of loaded TL is as same as that of the typical SPP TL, shown in Fig. 2(a), and the simulated S-parameters are shown in Fig. 2(b).

**Figure 3:**
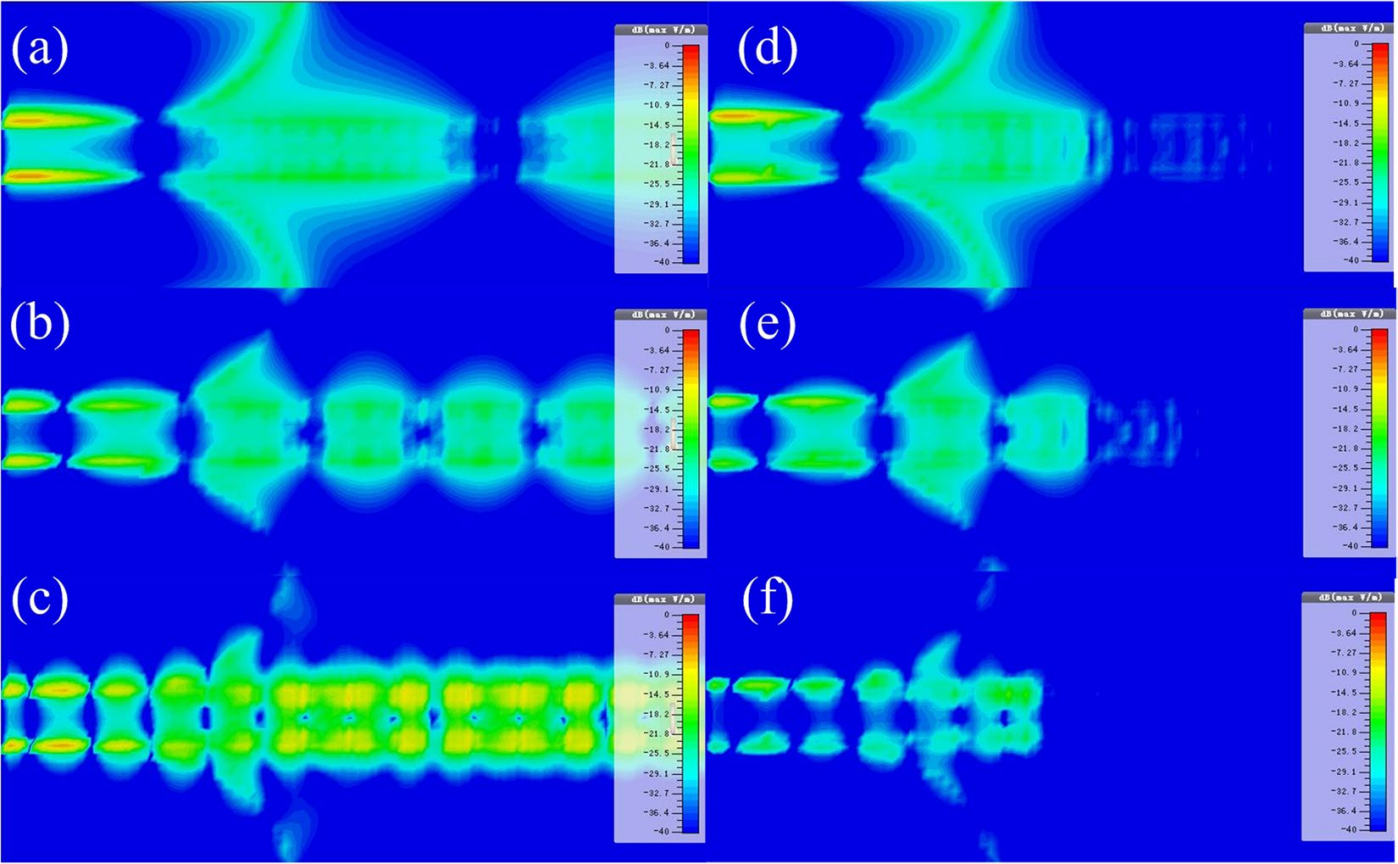
The simulated electric field above the SPP TL and the resistive loaded SPP TL at different frequencies (1 mm above the dielectric substrate). (**a**) 2 GHz (SPP TL). (**b**) 5 GHz (SPP TL). (**c**) 10 GHz (SPP TL). (**d**) 2 GHz (Loaded SPP TL). (**e**) 5 GHz (Loaded SPP TL). (**f**) 10 GHz (Loaded SPP TL).

The loaded TL should meet the following two conditions to estimate radiation loss of the input converter. First, energy on the corrugated metallic strip should be absorbed and converted to heat loss by resistors effectively in order to ensure that EM field along propagation direction decays fast enough and radiation from the corrugated metallic strip could be neglected; Second, the loaded resistors should guarantee matched impedance in order to make the mode converter work in the form of travelling wave. Electric field distribution above the typical SPP TL and the loaded TL at different frequencies are compared in Fig. 3. The similarity indicates that input converters of both TLs work in the same situation, and the fast attenuation of field along propagation direction ensures the correctness of the calculation of radiation loss from the input converter.

**Figure 4:**
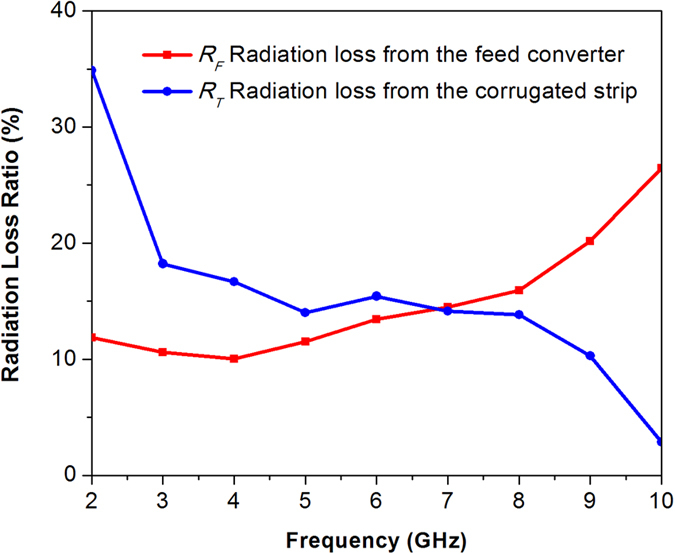
Radiation loss from different parts of the SPP TL.

Based on simulated far-field analysis, radiation loss from the input mode converter of the loaded TL is read off directly, and radiation loss from the corrugated metallic strip of finite length could be obtained by subtracting *R*_*F*_ from *R*_*RL*_. Radiation loss ratios from these two parts are shown in Fig. 4. According to the results, we deduce that radiation from the corrugated strip of finite length is the major part of radiation loss at low frequency band while input mode converter mainly causes radiation loss at high frequency band.

### Radiation loss from the corrugated metallic strip of finite length

Due to the periodic grooves, SPP TLs can support the surface wave mode like corrugated plane surfaces^26^. We use Floquet’s theory to analyze the SPP TL and find that unlike radiation from SPP antennas^27^, radiation from the corrugated metallic strip of finite length is not based on spatial harmonic waves. The distribution of current in Eq. (3) is periodic in x-axis with interval *P*.

3 $$J(x,t)=J(x){e}^{j2\pi ft}{e}^{-j{\beta }_{x}x}$$

Furthermore, using Floquet’s theory, we expand all spatial harmonic waves^28^ in Eq. (4).

4 $$J(x,t)={e}^{j2\pi ft}{\sum }_{n=-\infty }^{n=+\infty }{J}_{n}{e}^{\pm j\frac{2\pi n}{P}}{e}^{-j{\beta }_{x}x}$$

The phase constant of these spatial waves can be written as

5 $${\beta }_{xn}={\beta }_{x}\pm \frac{2\pi n}{P}$$

Because the size of grooves is far shorter than wavelength in air (*P* ≪ *λ*), spatial harmonic waves are all slow waves which radiate much less than fast waves. Therefore, *J*_*n*_ can be neglected for all *n* other than 0.

**Figure 5:**
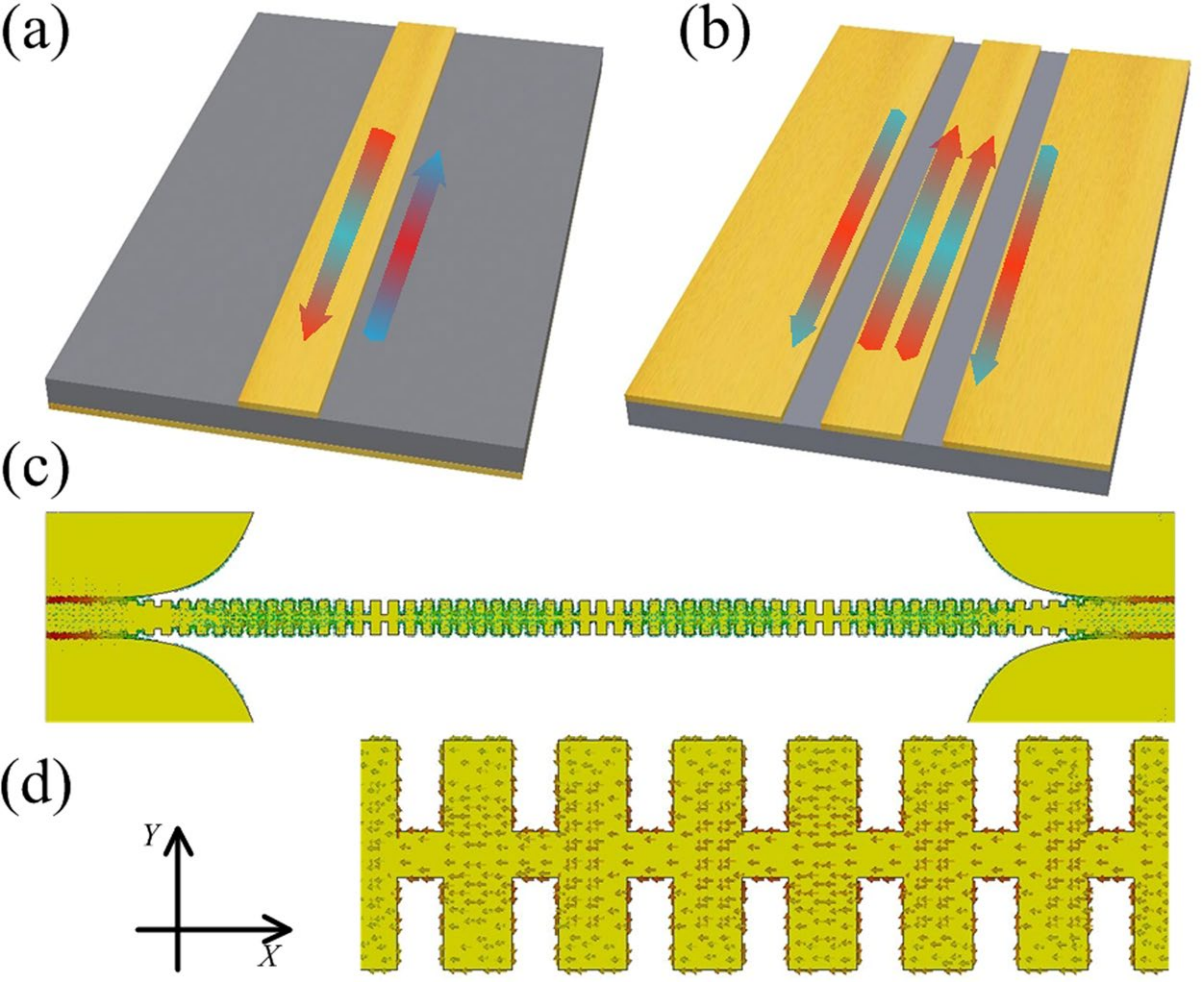
Current distribution on different TLs. (**a**) Current distribution of a microstrip. (**b**) Current distribution of a CPW. (**c**) Overview of currents on the corrugated strip *@f* = *2* *GHz*. (**d**) Detailed view on currents distribution of the SPP TL *@f* = *2* *GHz*.

Alternating current is one common source of radiation. Conventional multi-conductor TLs such as CPWs and microstrips radiate weakly because they always have pairs of opposing currents along propagation direction lying closely to each other as shown in Fig. 5(a and b). As a single conductor structure, the SPP TL is different in the term of the current distribution. Figure 5(c) shows the simulated distribution of current on the corrugated metallic strip. It can be observed that currents towards x-axis have no currents out-of-phase nearby. Radiation from these currents can’t be eliminated completely. These currents distribute mainly on the middle of the SPP TL, as shown in Fig. 5(d). Conversely, emissions from currents on the sides of grooves are eliminated because the width of grooves is far shorter than wavelength, and because the currents are in opposing phase. In addition, the symmetrical current distribution about the plane (xoz plane) normal to y-axis strengthens this cancellation. Hence, the main radiating elements of the SPP TL are currents distributing in x-axis on the middle of the corrugated strip.

**Figure 6:**
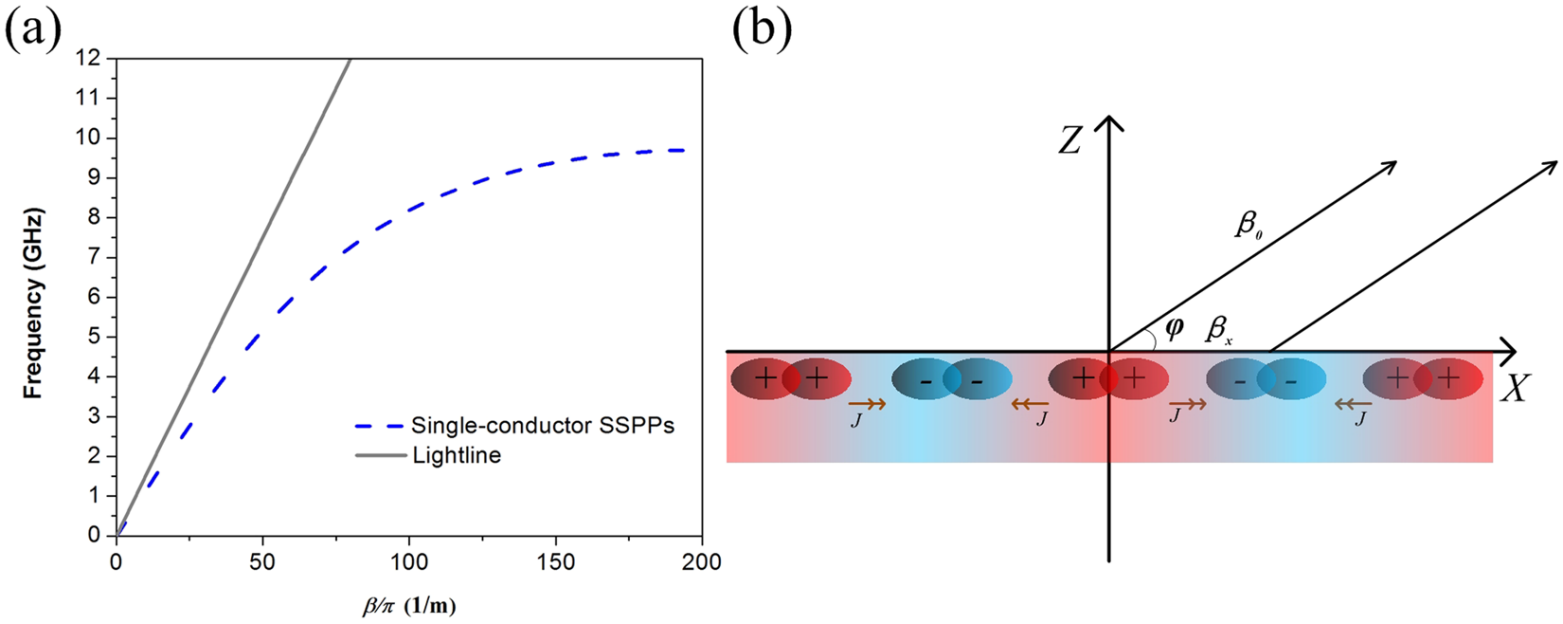
(**a**) Dispersion diagram of the SPP TL. (**b**) Radiation model based on currents along propagation direction.

To simplify the situation, three hypotheses are proposed. The first hypothesis is that radiation from currents in y-axis could be ignored due to the strong cancellation effect. The second hypothesis is that the current amplitude is constant along propagation. In addition, the phase constant of current is the same as that of the SSPPs as shown in Fig. 6(a). To verify our explanation of radiation loss from finite corrugated metallic strip, a simplified model based on vector currents is established as shown in Fig. 6(b).

The SPP TL’s midpoint is set as the origin of the coordinate system. Eq. (6) shows a current element along x-axis in a form of travelling wave.

6 $${J}_{x}={J}_{0}\times {e}^{-j{\beta }_{x}x}$$

Based on Eq. (6), normalized radiation pattern can be obtained as Eq. (7). Eq. (7) consists of three parts. The first part *C* represents the constant proportional to the max radiation gain. The second part (sin*φ*)^2^ represents the radiation characteristic of a current element. The last part determines radiation lobes. If the length of mode converter (*T*) is negligible compared with the length of the total SPP TL (*T* ≪ *L*), then effective length *L*_*e*_ equals *L*. If not, the effective length should be approximated as *L*_*e*_ = *L* − 2·0.5·*T*.

7 $$D=C\times {(\sin \phi )}^{2}\times {(\frac{\sin [({\beta }_{0}\cos \phi -{\beta }_{x})\times {L}_{e}/2]}{{\beta }_{0}\cos \phi -{\beta }_{x}})}^{2}$$

Eq. (7) shows the difference of radiation between fast-wave TLs and slow-wave TLs. When |*β*_*x*_| < |*β*_0_|, the equation approaches a maximum value when (${\beta }_{0}\,\cos \,\phi -{\beta }_{x}=0$). The angle at this point determines the position of the main lobe. When |*β*_*x*_| > |*β*_0_|, the denominator never equals zero, meaning that the main lobe gain of slow-wave TLs is much smaller compared to that of fast-wave TLs. When the length, angle and phase constant make the numerator zero, the EM field vectors in the far field from all the currents are canceled out. At this angle, no radiation exists because of the elimination effect. Nevertheless, it is impossible to make the numerator zero at any angle, so slow-wave TLs radiate except for certain special angles. These special angles represent null radiation directions in radiation pattern, and there is always a radiation lobe between any two adjacent special angles.

**Figure 7:**
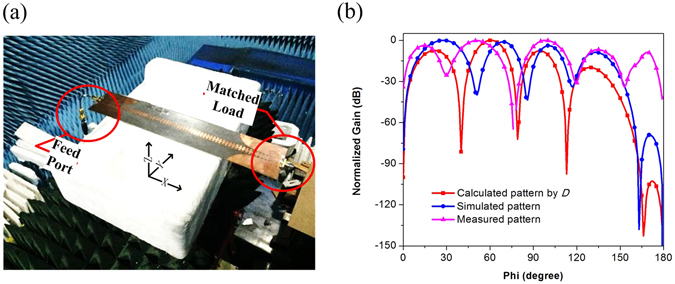
(**a**) Measurement environment in an anechoic chamber. (**b**) Normalized radiation patterns obtained by three different methods.

Since radiation loss from corrugated metallic strip plays a key role at low frequency band, normalized radiation patterns obtained by Eq. (7), by simulation, and by far-field measurement are shown in Fig. 7. The TL was placed on a turntable, one of two ports was used for feeding and the other port was connected to a matched load to ensure the SPP TL works in a mode of travelling wave. It can be observed that the SPP TL radiates towards almost all directions. The difference of patterns is mainly brought by the coaxial cable connector from experiment setup. The similarity of radiation patterns obtained by three methods, especially the same position of lobes, vindicates our explanation.

In addition, the radiation pattern, especially the distribution of lobes is similar to that of a long wire antenna^28–30^. Radiation lobes of both a long wire antenna and the SPP TL distribute symmetrically about the yoz plane and the xoz plane. Furthermore, radiation patterns have diminishing radiation lobes as the angle increasing from 0 degree to 180 degrees, and the smallest lobe lies in the direction opposite to the propagation.

**Figure 8:**
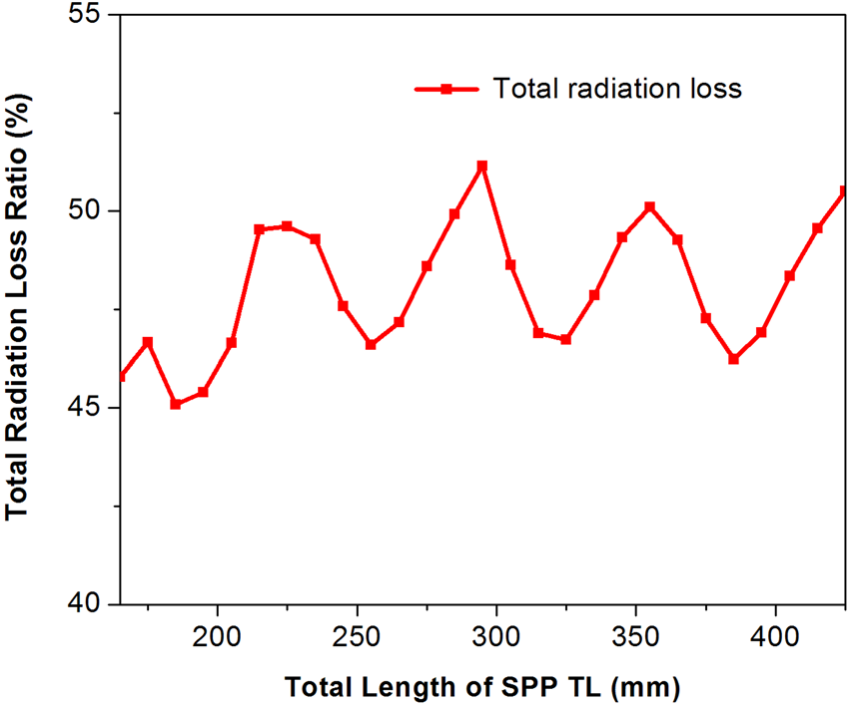
Relationship between radiation loss and length.

Measurements of transmission characteristics of SPPs TLs with different length were presented in 18. Based on the phenomenon that transmission efficiency was not related to the total length of TLs, Kianinejad, A. *et al*. thought radiation loss mainly comes from mode converters. A similar phenomenon is observed again, as shown in Fig. 8, when we investigate the change of total radiation loss with the total length of the SPP TL at 2 GHz. Radiation loss fluctuates slightly with increasing of the length, instead of being a constant or changing linearly with length. In this section, this paper explains why it may be inappropriate to think the radiation loss is proportional to the total length of the SPP TL, and estimates radiation loss from corrugated strip of finite length from a different aspect.

The effective radiation section (ERS) theory was presented to study positions of side lobes from leaky wave antennas in recent researches^24, 25^. But the radiation loss, in other words, the radiation efficiency has not been discussed. By taking the average value of ERS at angles from 0 to 180 degrees, the concept of average radiation length (ARL) is proposed to discuss radiation efficiency from the finite corrugated metallic strip. Note that the definition of axes in this paper is different from that in 24 and 25, some important formulas need to be rewritten before calculating ARL.

The length of ERS (*L*_*ERS*_) is defined by the following expressions^24, 25^. At a given angle, the emissions from two segments with length *S* will be cancelled out completely. *S* is defined as Eq. (8), relating to *β*_0_ the phase constant of free space and *β*_*x*_ the phase constant along the TL.

8 $$S(\phi )=\frac{\pi }{|{\beta }_{0}\,\cos \,\phi -{\beta }_{x}|}$$

The length of ERS is obtained in Eq. (9) where *N* is the number of segment pairs with spacing *S*. *Le* is the effective length used in Eq. (7).

9 $${L}_{ERS0}(\phi )=\frac{{L}_{e}-2N(\phi )S(\phi )}{2}$$

Eq. (9) should be modified as^25^

10 $${L}_{ERS}(\phi )=\{\begin{array}{cc}{L}_{ERS0}(\phi ) & ,2{L}_{ERS0}\le S(\phi )\\ S(\phi )-{L}_{ERS0}(\phi ) & ,2{L}_{ERS0} > S(\phi )\end{array}$$

Furthermore, we propose ARL to estimate radiation loss from slow wave TL of finite length.

11 $$ARL=\frac{1}{\pi }{\int }_{0}^{\pi }{L}_{ERS}(\phi )\cdot d\phi $$

**Figure 9:**
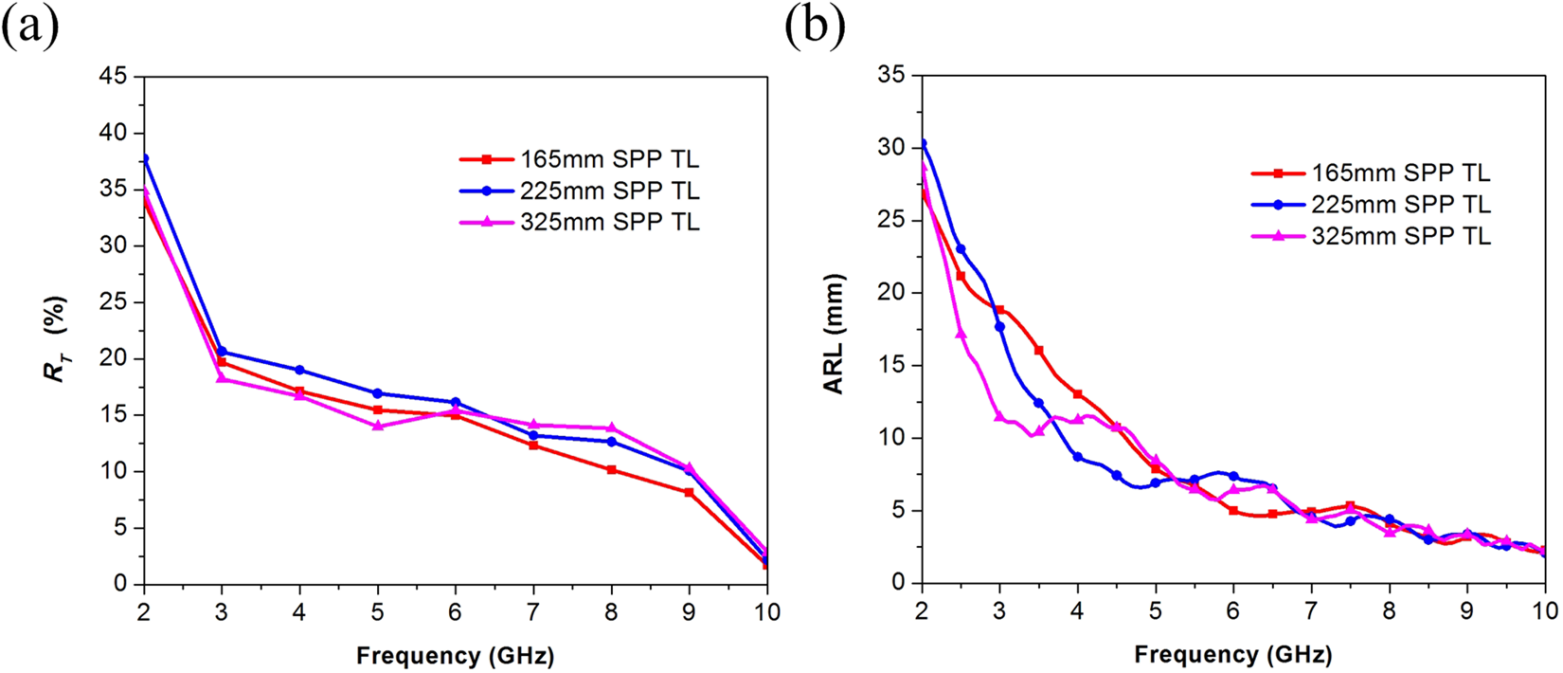
(**a**) Radiation loss from corrugated metallic strip. (**b**) Average radiation length (ARL).

Radiation loss from finite corrugated metallic strip is independent of the total length of TLs, and seems proportional to ARL since the radiation of finite slow wave structure comes from ERS, a function of angles^24, 25^. More evidence is found to verify our explanation. Simulated total radiation loss *R*_*RL*_ is calculated by far-field analysis, and after subtracting radiation loss from the feed converter *R*_*F*_, radiation loss from corrugated strip *R*_*T*_ is distinguished as shown in Fig. 9(a). ARL of three SPPs TLs of different lengths are shown in Fig. 9(b).

### Solution to suppress radiation loss

**Figure 10:**
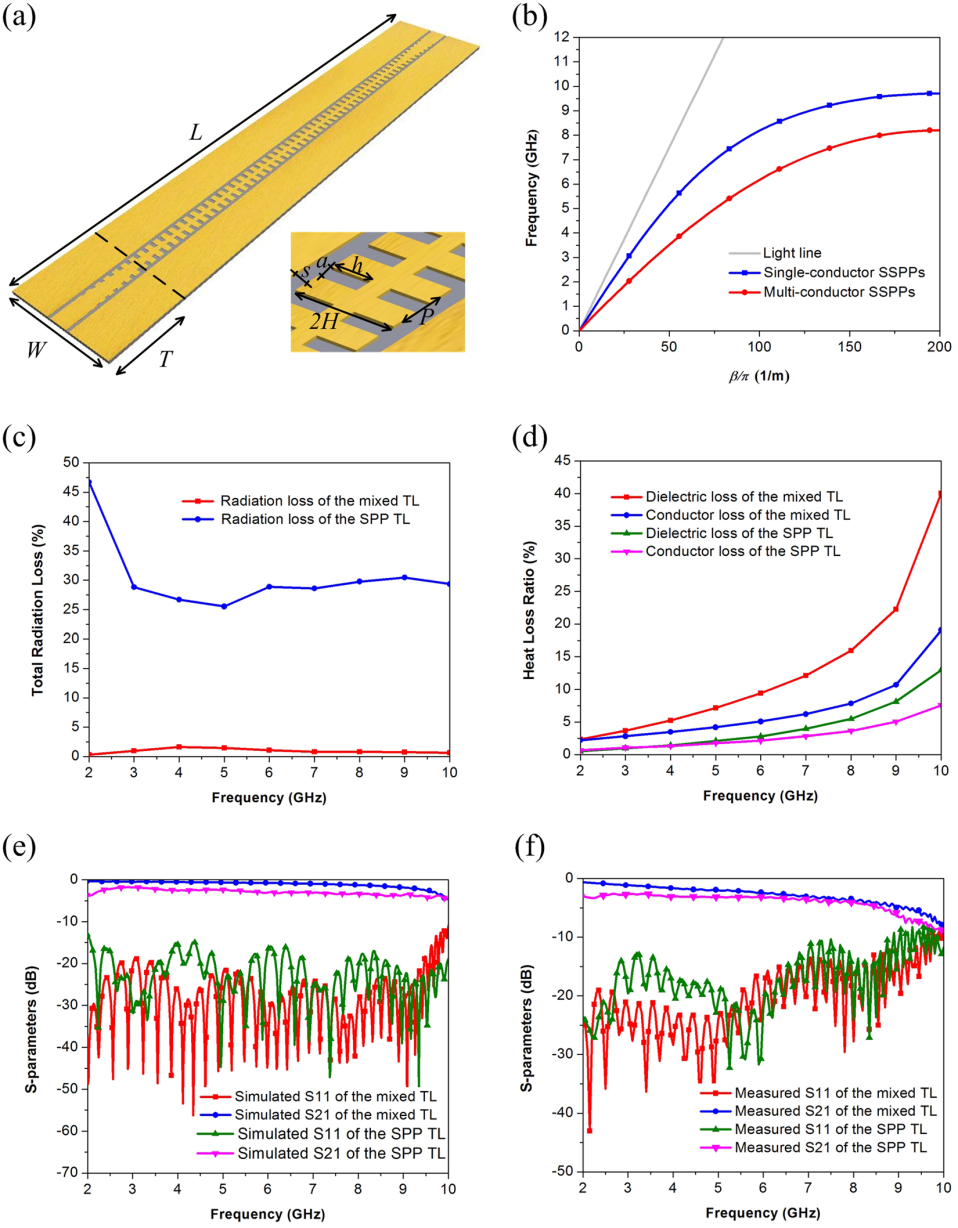
(**a**) The configuration of the mixed TL in which *L* = 325 mm, *W* = 60.8 mm, *T* = 60 mm and the F4b substrate is 0.5 mm thick. The inset picture shows the structure of the unit cell, in which *P* = 5 mm, *a* = 2 mm, *h* = 4 mm, *H* = 5 mm, *S* = 0.4 mm. (**b**) Dispersion diagrams. (**c**) Total radiation loss. (**d**) Dielectric loss and conductor loss. (**e**) Simulated S-parameters. (**f**) Measured S-parameters.

In order to suppress radiation loss, it is necessary to establish currents of opposing phase along TLs. This paper proposes a mixed TL shown in Fig. 10(a). The multi-conductor structure consists of two ground planes of CPW and corrugated metallic strip. The mixed TL has stronger slow-wave effect than SPP TLs with units of the same size, as shown in Fig. 10(b). It may be controversial to call the mixed TL structure a spoof SPP TL, but there have been reports about SPP filters with similar structures^31^. Due to the existence of currents out-of-phase along propagation, radiation loss of the mixed TL decreases dramatically, as compared in Fig. 10(c). The radiation loss of the mixed transmission line is less than 5% while the radiation loss of the SPP TL is more than 25%. The mixed TL solves the problem of radiation loss which is the main drawback of traditional SPP TLs at low frequency band, but may bring more dielectric loss and conductor loss at high frequency band, as show in Fig. 10(d). The simulated and measured S-parameters of the mixed TL and the SPP TL are respectively shown in Fig. 10(e and f). Both simulated and measured S_21_ curves of the mixed TL lie above that of the SPP TL, indicating the mixed TL has better transmission efficiency.

## Discussion

Many researchers regarded SPP TLs as low-loss TLs without taking radiation loss into consideration, although total loss of a TL may include four parts: reflection loss, conductor loss, dielectric loss, and radiation loss. Generally, reflection loss is mainly caused by mismatch, which can be minimized by carefully designed transition structure. Conductor loss comes from metal resistance. Dielectric loss depends on the loss tangent of substrates. Radiation loss is usually related to the structure of TLs, which reduces the transmission efficiency and causes interference signal. We investigate radiation loss from SPP TLs that is usually ignored in pervious papers. Over 25% of the total energy input into the SPP TL is radiated at the full frequency band. Due to the input mode converter and the special current distribution, SPP TLs radiate inevitably. Radiation from the input mode converter and that from corrugated metallic strip of finite length is analyzed separately. Proposing the concept of ARL in this paper, we explain why radiation loss is not proportional to the total length of TLs. The key method to suppress radiation loss is to construct pairs of currents out-of-phase. Based on the method, a mixed structure is proposed, which provides larger phase constant and simultaneously reduces radiation loss. Hopefully, it will become one of the candidates of slow-wave TLs.

## Methods

Numerical simulations were performed by the commercial software, CST Microwave Studio. The far field radiation pattern was measured in an anechoic chamber. And the S-parameters were measured by ROHDE&SCHWARZ ZVA40 vector network analyzer.
